# Improved application of the electrophoretic tissue clearing technology, CLARITY, to intact solid organs including brain, pancreas, liver, kidney, lung, and intestine

**DOI:** 10.1186/s12861-014-0048-3

**Published:** 2014-12-21

**Authors:** Hyunsu Lee, Jae-Hyung Park, Incheol Seo, Sun-Hyun Park, Shin Kim

**Affiliations:** Department of Anatomy, Keimyung University School of Medicine, 1095 Dalgubeoldae-Ro, Dalseo-Gu, 704-701 Daegu South Korea; Department of Physiology, Keimyung University School of Medicine, 1095 Dalgubeoldae-Ro, Dalseo-Gu, 704-701 Daegu South Korea; Department of Microbiology, Keimyung University School of Medicine, 1095 Dalgubeoldae-Ro, Dalseo-Gu, 704-701 Daegu South Korea; Department of Immunology, Keimyung University School of Medicine, 1095 Dalgubeoldae-Ro, Dalseo-Gu, 704-701 Daegu South Korea; Department of Microbiology and Immunobiology, Harvard Medical School, Division of Neuroscience New England Primate Research Center, 1 Pine Hill Drive, Southborough, 01772 MA USA

**Keywords:** CLARITY, Brain, Nervous system, Electrophoretic tissue clearing, 3D Reconstruction, Purkinje layer

## Abstract

**Background:**

Mapping of tissue structure at the cellular, circuit, and organ-wide scale is important for understanding physiological and biological functions. A bio-electrochemical technique known as CLARITY used for three-dimensional anatomical and phenotypical mapping within transparent intact tissues has been recently developed. This method provided a major advance in understanding the structure-function relationships in circuits of the nervous system and organs by using whole-body clearing. Thus, in the present study, we aimed to improve the original CLARITY procedure and developed specific CLARITY protocols for various intact organs.

**Results:**

We determined the optimal conditions for reducing bubble formation, discoloration, and depositing of black particles on the surface of tissue, which allowed production of clearer organ images. We also determined the appropriate replacement cycles of clearing solution for each type of organ, and convincingly demonstrated that 250–280 mA is the ideal range of electrical current for tissue clearing. We then acquired each type of cleared organs including brain, pancreas, liver, lung, kidney, and intestine. Additionally, we determined the images of axon fibers of hippocampal region, the Purkinje layer of cerebellum, and vessels and cellular nuclei of pancreas.

**Conclusions:**

CLARITY is an innovative biochemical technology for the structural and molecular analysis of various types of tissue. We developed improved CLARITY methods for clearing of the brain, pancreas, lung, intestine, liver, and kidney, and identified the appropriate experimental conditions for clearing of each specific tissue type. These optimized methods will be useful for the application of CLARITY to various types of organs.

**Electronic supplementary material:**

The online version of this article (doi:10.1186/s12861-014-0048-3) contains supplementary material, which is available to authorized users.

## Background

Tissue clearing technologies, such as CLARITY [1] and perfusion-assisted agent release in situ (PARS) [2], which create optically transparent and macromolecule-permeable images, have provided a major advance in the imaging of biological systems. These methods improve tissue permeability by replacing the lipid bilayer of plasma membranes with a nanoporous hydrogel. Unlike mechanical micro-dissection methods, which aggravate deformation of tissue structure alongside of micro-dissection, CLARITY preserves the intact structure of brain organs, allowing the tracing of neurite’ projections, and the three-dimensional (3D) and topological reconstruction of traced neurons [1]. PARS can also produce macromolecule permeability and optical transparency in the brain and other organs [2].

Obtaining detailed system-wide informations of organs using a general optical microscope is formidable. For example, single-photon microscopy provides a maximum of 50 μm imaging depth below the organ surface, while even well-optimized two-photon microscopy cannot image deeper than approximately 800 μm [3]-[5]. However, in various intact organs, CLARITY or PARS can overcome these limitations to permit enhanced viewing of organ structures at greater depths [1],[2], providing access to integrated structural and molecular information from the brain and other intact biological systems [1],[2]. In particular, CLARITY provides an accelerated rate of un-dissected tissue clearing in the brain through applied electric force, and also avoids tissue damage during clearing [1],[2],[6].

In the present study, we evaluated whether CLARITY could be effectively applied to other intact organs from the adult mouse (12 weeks-old), including the pancreas, liver, lungs, intestines, and kidneys, as well as the whole brain. Although, this technique has been applied to various cerebral regions of the brain [1], herein, we particularly focused on the Purkinje cell layer of the cerebellum. Furthermore, we examined the structural integrity of cell-vasculature relationships in the pancreatic tail region using single-photon microscopy (0.7 mm). This approach allowed us to obtain a detailed, objective picture of the complexity of the islet vascular system. These data suggest that organ clearing is useful for examining the physiology and pathophysiology of the vascular system as well as other organs.

## Methods

### Experimental animals

All animal experimental procedures were conducted in accordance with the guidelines of the University Committee on Animal Resources at Keimyung University (Approval No. KM-2014-20R1).

### CLARITY applied to the mouse brain

Adult mice (12 weeks old) were anesthetized using a combination of tiletamine-zolazepam-xylazine and perfused transcardially with 30 mL of ice cold 1X phosphate-buffered saline followed by 30 mL of ice cold hydrogel solution with a mixture of 4% PFA, 4% acrylamide, 0.05% bis-acrylamide, 0.25% VA044 in PBS. Organs were extracted and incubated in the same solution at 4°C for 7 days. The solution temperature was then increased to 37°C to initiate polymerization. After 3 h at 37°C, hydrogel-embedded organs were placed in an electrophoretic tissue clearing (ETC.) chamber. While sodium borate buffer (200 mM, pH 8.5) containing 4% SDS (the clearing solution) was circulated through the chamber, 250–280 mA was applied across the organs at 42°C for 2–4 weeks. The solution circulation velocity was 28 L/min, and the volume of clearing solution was 10 L. The clearing solution replacement cycle is described in Table 1. After clearing, the organs were incubated in PBS at 37°C for 4 days to remove SDS. 3-mm-thick horizontal blocks of mouse brain were cleared by electrophoresis for 3 days as described in experimental conditions. The detailed experiment processes were as previously reported [1].

**Table 1:** **Optimized ETC. conditions for various tissues**

Organ	Optimized ETC. time (days)	Constant current (mA)	Varying voltage (V)	Replacement interval of clearing solution (days)
Brain	Whole brain: 12-16	280	22 ~ 26	5
	3-mm-thick-block: 3			N/A
Pancreas	8-12	250		7
Kidney	18-22	280		7
Liver	18-22	280		7
Intestine	8-12	250		7
Lung	13-17	250		7

n = 30 for optimization of each organ clearing. ETC., electrophoretic tissue clearing; mA, milliampere; V, voltage; N/A, not available.

### Immunostaining of CLARITY - processed mouse brain and pancreas

To prepare mouse brain for immunostaining, hydrogel-embedded and clarified brains were cut into 3-mm-thick horizontal blocks using a mouse brain matrix (Ted pella). For axon staining, the clarified brain was incubated at 37°C for 3 weeks in 0.1% triton X-100, and 1 M sodium borate buffer, pH 8.5, with an anti-tau1 antibody (1:50; Cat# MAB3420, Millipore Corp., Bedford, MA, USA). For pancreatic blood vessels, the clarified pancreas was incubated at 37°C for 2 weeks in 0.1% triton X-100 and 1 M sodium borate buffer, pH 8.5, with anti-α-smooth muscle actin (1:50; Cat# ab5694, Abcam, Cambridge, UK). The brain and pancreas were washed at 37°C for 1 week in 0.1% triton X-100 and 1 M sodium borate buffer, pH 8.5. The samples were then incubated at 37°C for 2 weeks in 0.1% triton X-100 and 1 M sodium borate buffer, pH 8.5, with a goat anti-rabbit-AlexaFluor488 secondary antibody (1:100; Cat# A11029, Invitrogen). For nuclear staining, the clarified pancreas was incubated at 37°C for 30 min in DAPI (0.1 μg/mL, Cat# D9542, Sigma-Aldrich, St. Louis, MO, USA).

### Imaging of CLARITY - processed mouse brain and pancreas

For imaging the structures of brain and pancreas, the clarified brain and pancreas were incubated in FocusClear, a water-based immersion medium, for 4 days. The brain and pancreas were then enclosed between two coverglass-bottom petri dishes. The brain and pancreas were imaged (Z-stack volume, 110–650 μm) using confocal laser scanning microscopy (LSM 5 EXCITER; Carl Zeiss, Jena, Germany) at excitation wavelengths of 488 nm with a C-Apochromat × 40 objective (1.2 numerical aperture). Three-dimensional reconstruction was generated using LSM5 EXCITER software (Carl Zeiss). To extract the pancreatic microvasculature images, we carefully traced the anti-α-smooth muscle actin profiles using the ImageJ plug-in Single Neurite Tracer [7].

## Results

### Electrical current conditions related to enhancing CLARITY

To clear the hydrogel-embedded tissues after completing the polymerization, an electric field was applied to the tissues in an ETC. chamber which was built as described previously [1]. Although the previous study recommended using high voltage and temperature to clear tissue, the high constant voltage caused bubble formation, discoloration and/or the deposition of black particles in tissue samples (Additional file 1). Specifically, the constant voltage created a high current thus damaging the tissue. The previous study also recommended low voltage (V) (10 - 40 V) for ETC. [1], but this condition was shown to be ineffective in applying to other types of tissue as well as the whole brain. Therefore, we determined the optimal range of electrical current condition for clearing tissues.

**Figure 1:**
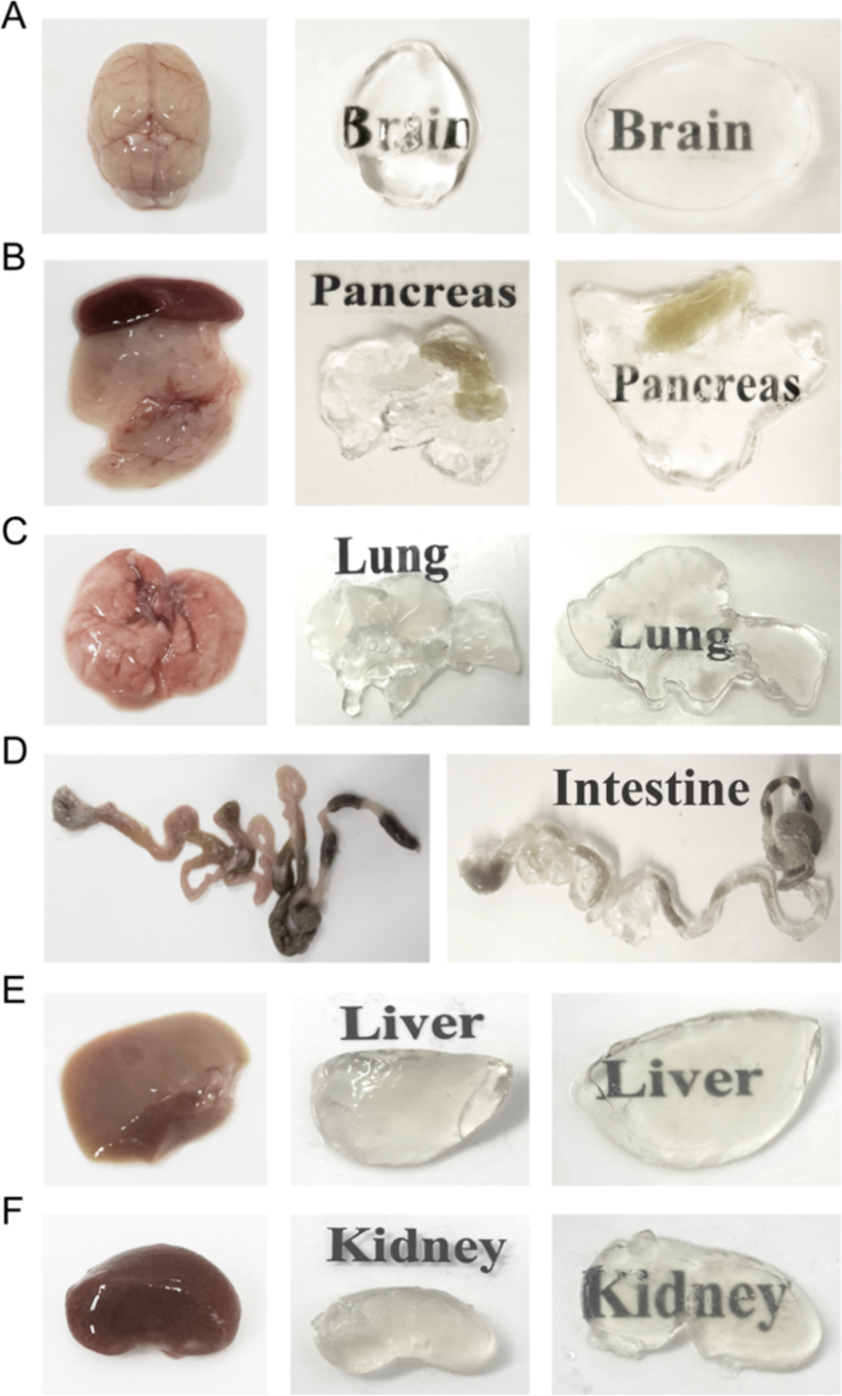
**CLARITY of intact adult mouse tissues. In adult mouse tissues (12 weeks old), imaging was performed before and/or after CLARITY. (A)** Brain. **(B)** Pancreas. **(C)** Lung. **(D)** Intestine. **(E)** Liver. **(F)** Kidney.

We found that for brain samples, 280 mA was the optimal current without causing any damage. When the constant current of 280 mA was maintained across the sample and the clearing solution was replaced three times over 2 weeks, the sample was completely cleared (Figure 1A). Additionally, it was determined that at a constant electrical current condition, the measured voltage between the platinum wires of chamber should reach at least 20–30 V for successful tissue clearing.

We then investigated the optimal electrical current conditions for the clearing of mouse pancreas and lung tissues. When we identified the appropriate clearing conditions of the tissues, 250 mA is the appropriate current because these tissues are softer than brain tissue. The pancreas sample was cleared by applying 250 mA and replacing the clearing solution two times over 2 weeks (Figure 1B). Lung tissue was cleared by applying 250 mA and replacing the clearing solution three times over 3 weeks (Figure 1C). We tested additional solid organs for clearing under these conditions. The mouse intestine sample was cleared by applying 250 mA and replacing the clearing solution three times over 3 weeks (Figure 1D). Finally, mouse liver and kidney samples were cleared by applying 280 mA and replacing the clearing solution four times over 4 weeks (Figure 1E, F).

### Imaging of structures in the adult mouse brain and intact pancreas

**Figure 2:**
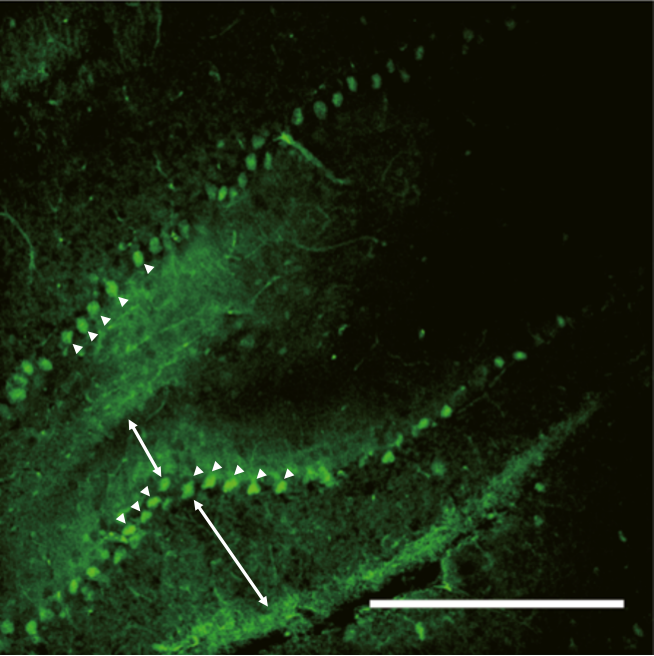
**Cerebellar Purkinje layer imaging in the adult mouse brain.** The Purkinje layer of cerebellar region form the clarified mouse brain was immunostained with tau (green). Scale bar, 500 μm. Purkinje cell (arrow heads), granular layer (upper, a double-headed arrow), and molecular layer (lower, a double-headed arrow). Representative data were chosen from five independent experiments.

**Figure 3:**
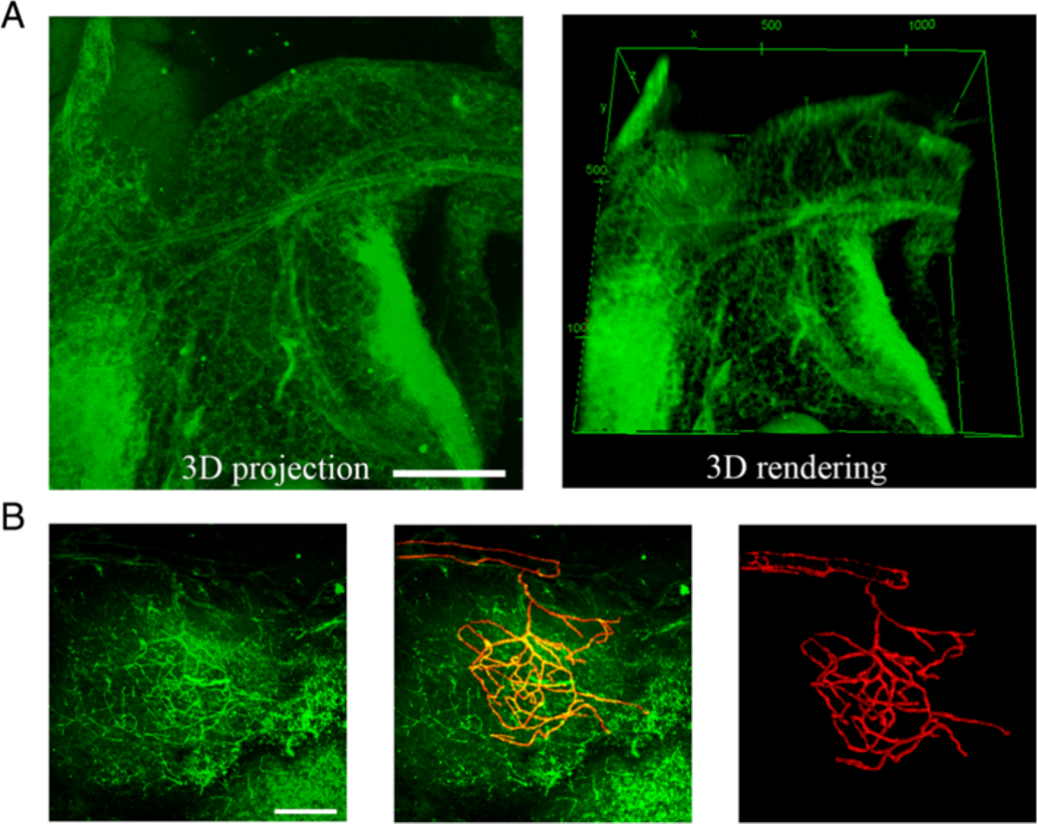
**Pancreatic vessel imaging in the intact adult mouse pancreas.** In adult mouse tissues (12 weeks old), imaging was performed after CLARITY. **(A)** Three-dimensional (3D) projection (left panel) and rendering (right panel) of clarified mouse pancreas without capillary immunostained for α-smooth muscle actin (green). Scale bar, 300 μm (Additional files 3 and 4). **(B)** Three-dimensional projection (left panel) clarified mouse pancreas with capillary immunostained for α-smooth muscle actin (green). Scale bar, 200 μm. Merged image with manually traced (green, middle panel) and 3D rendering extracted capillary (right panel). Representative data were chosen from five independent experiments.

**Figure 4:**
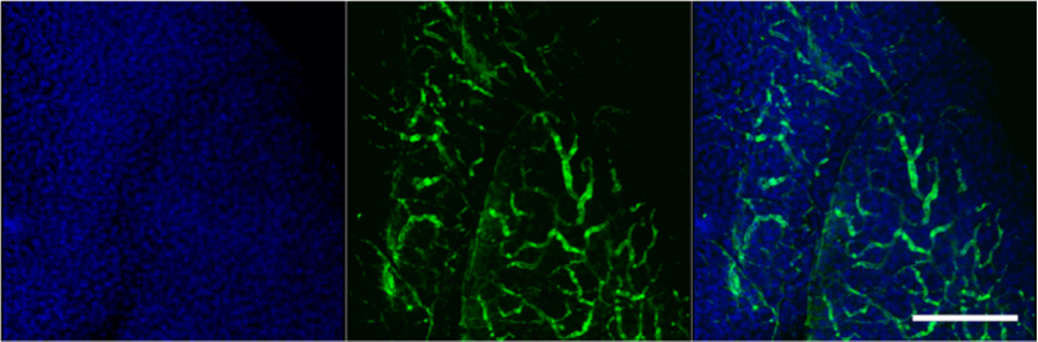
**Pancreatic vessel imaging in the intact adult mouse pancreas.** Three-dimensional projection (left panel and middle panel) clarified mouse pancreas with nuclear stained for DAPI (blue) and vascular immunostained for α-smooth muscle actin (green). Merged image with 3D rendering nuclei (blue, left panel) and 3D rendering vessels (green, middle panel). Scale bar, 200 μm. Representative data were chosen from five independent experiments.

We performed immunostaining in adult mouse brain using a microtubule-associated protein tau-antibody. Using confocal images, tau-stained axon fiber was confirmed in the hippocampal region (Additional file 2) and the Purkinje layer of cerebellum (Figure 2). As shown in Additional file 2, the hippocampal region was reconstructed in 3D stacks of images (Z-stack volume, 650 μm). Moreover, the Purkinje layer of cerebellum was reconstructed in 3D stacks of images (Z-stack volume, 210 μm) (Additional file 3 and Additional file 4). Furthermore, we performed imaging in pancreas sample with an α-smooth muscle actin-antibody which is the marker for blood vessels including capillary vessels [8]. We could identify stained vessels in randomly selected regions of distal pancreas in optically transparent pancreas (Figure 3A, Additional files 5, 6, 7, 8 and 9). The vessel and pancreatic region was reconstructed in 3D stacks of images (Z-stack volume, 650 μm). To reconstruct the 3D morphology of the pancreatic capillaries, we traced the profile of the α-smooth muscle actin and extracted the image of the pancreatic capillary using ImageJ (Figure 3B, Additional file 10). To identify the structural integrity of cells-vasculature relationships in distal region of the pancreas, we fluorescent stained the cleared pancreas samples with an α-smooth muscle actin-antibody and DAPI, which binds strongly to A-T rich regions in DNA. We could identify stained vessels and pancreatic cellular nuclei in randomly selected regions of the distal pancreas in optically transparent pancreas (Z-stack volume, 110 μm) (Figure 4, Additional file 11).

## Discussion

Using our optimized CLARITY protocol, we were able to successfully image various mouse whole organs, including the brain and the pancreas. Imaging of blood vessels in the pancreas allowed the general vessel pattern to be determined under normal conditions. This clearing technique may be useful in future studies related to pancreatic disease, of which there have been limited images of blood vessels or other structures. We were also able to identify tau-stained neurons in the Purkinje layer of the cerebellum. Further, we determined the optimal electrical current conditions for clearing of specific organs without bubbling, discoloration, or accumulation of black particles in tissue samples during ETC.

A previous study reported that CLARITY is an effective imaging technique for mapping the nervous system in the brain [1]. However, as bubbling, discoloration, and the appearance of black particles were observed in some samples, further investigation to determine optimal clearing conditions were recommended [1]. In the present study, we found that the range of voltage (10–60 V) was too broad to be practical. Thus, we determined the appropriate experimental conditions for a variety of tissues to produce more clear images and minimize tissue damage, and found an optical electrical current of 250–280 mA (Table 1). Yang et al. reported that PARS method, a method that provides tissue clearance of intact whole-organisms, could provide access to integrated structural and molecular information from the brain and other intact biological systems [2]. Although, organs with a higher cell density (e.g., kidney and liver) required up to 22 days for tissue clearance, using our appropriate experimental conditions, we demonstrated that CLARITY with ETC. could be effectively applied to organs such as the pancreas, liver, lung, kidney and intestine without dissecting the organs. Although Yang et al. introduced a protocol for passive tissue clearing passive clarity technique (PACT), the method required 3 weeks for clearing of un-dissected, whole adult mouse brain without electrophoretic instrumentation (passive CLARITY) [2]. In our study, although longer times were required to clear tissues than those previously reported [1], our methods provide an important advance for the CLARITY technique, allowing application to a variety of other types of organ.

A major finding of our study was that CLARITY could be successfully applied to intact mouse organs including the brain, pancreas, liver, lung, kidney, and intestine (Figure 1). For example, we were able to visualize intact structures and reconstruct the pattern of blood vessels with 3D reconstruction in the optically transparent pancreas. The vascular pattern of the intact pancreas is poorly understood as it is challenging to trace whole vessels using general imaging with 3D reconstruction. Thus, our modified technique may be particularly useful for studying the vascular system in pancreatic function and homeostasis [4],[9]. We also demonstrated the 3D reconstruction of the Purkinje layer and adjacent layers in the cerebellar region, suggesting that this modified technique can be applied to the understanding of human disease related to Purkinje cell.

In the present study, we have optimized the CLARITY method for use in tissue clearing of the pancreas, liver, lung, kidney and intestine. In particular, we confirmed that the clarified pancreas allows imaging of the partial vascular structure using single-photon microscopy. Further, we provide the first evidence of 3D imaging of the Purkinje layer of cerebellar region and the cell-vasculature relationships. Overall, our data suggest that CLARITY may be useful for examining a variety of biological tissues.

## Conclusions

The technique known as CLARITY used for 3D anatomical and phenotypical mapping within transparent intact tissues has been recently developed. This enhanced technology provides an innovative method for the acquisition of integrated structural and molecular information from the various intact organs across species. The results of this study show that the applicability of CLARITY with ETC. to other types of organ including the pancreas, lung, intestine, liver, and kidney. During establishing the suitable protocols for each intact organ, we encountered difficult situations such as bubble formation, discoloration, and the depositing of block particles on the surface of tissue. However, we overcame the experimental troubles and elaborated the optimal conditions, thus producing more clear images. Importantly, we determined that the supply of 250–280 mA is the ideal range of electrical current for tissue clearing. Moreover, this study demonstrated for the first time that the Purkinje layer of cerebellar region and the cells-vasculature relationships of pancreatic region were reconstructed in 3D stacks of image (Z-stack volume, 650 μm). Finally, having obtained the desired image using CLARITY, we concluded that this method can be applied to various organs besides mouse brain and it would be helpful for other researchers who want to conduct their own CLARITY research.

## Additional files

## Electronic supplementary material

Additional file 1: Figure that Problems of high voltage condition (30 voltage, 42°C) during CLARITY. (A) Bubble formation and discoloration in brain. (B) Deposit of black particle in brain. (TIFF 12 MB)

Additional file 2: Figure that three-dimensional (3D) projection (left panel) and rendering (right panel) of clarified mouse brain immunostained for tau (green). Scale bar, 300 μm. (TIFF 6 MB)

Additional file 3: **Movie that 3D immunohistological visualization of the Purkinje layer of cerebellar region in Figure** 2**.** (MP4 9 MB)

Additional file 4: **Movie that 3D immunohistological visualization of the Purkinje layer of cerebellar region in Figure** 2**.** (MP4 3 MB)

Additional file 5: Movie that 3D immunohistological visualization of alpha-smooth muscle actin in mouse pancreas. (MP4 5 MB)

Additional file 6: Movie that 3D immunohistological visualization of alpha-smooth muscle actin in mouse pancreas. (MP4 6 MB)

Additional file 7: Movie that 3D immunohistological visualization of alpha-smooth muscle actin in mouse pancreas. (MP4 8 MB)

Additional file 8: Movie that 3D immunohistological visualization of alpha-smooth muscle actin in mouse pancreas. (MP4 7 MB)

Additional file 9: Movie that 3D immunohistological visualization of alpha-smooth muscle actin in mouse pancreas. (MP4 5 MB)

Additional file 10: **Movie that 3D visualization of extracted capillary in Figure** 3**B.** (MP4 2 MB)

Additional file 11: **Movie that 3D visualization of vessels and extracted capillary in Figure** 4**.** (MP4 2 MB)

## Abbreviations

mA: milliampere
ETC.: Electrophoretic tissue clearing
PARS: Perfusion-assisted agent release in situ
PACT: Passive clarity technique
