# Supplementary figures and images for: Improved application of the electrophoretic tissue clearing technology, CLARITY, to intact solid organs including brain, pancreas, liver, kidney, lung, and intestine
Source: BMC Dev Biol. 2014 Dec 21;14:48. doi: 10.1186/s12861-014-0048-3 (PMC4281481; doi:10.1186/s12861-014-0048-3)

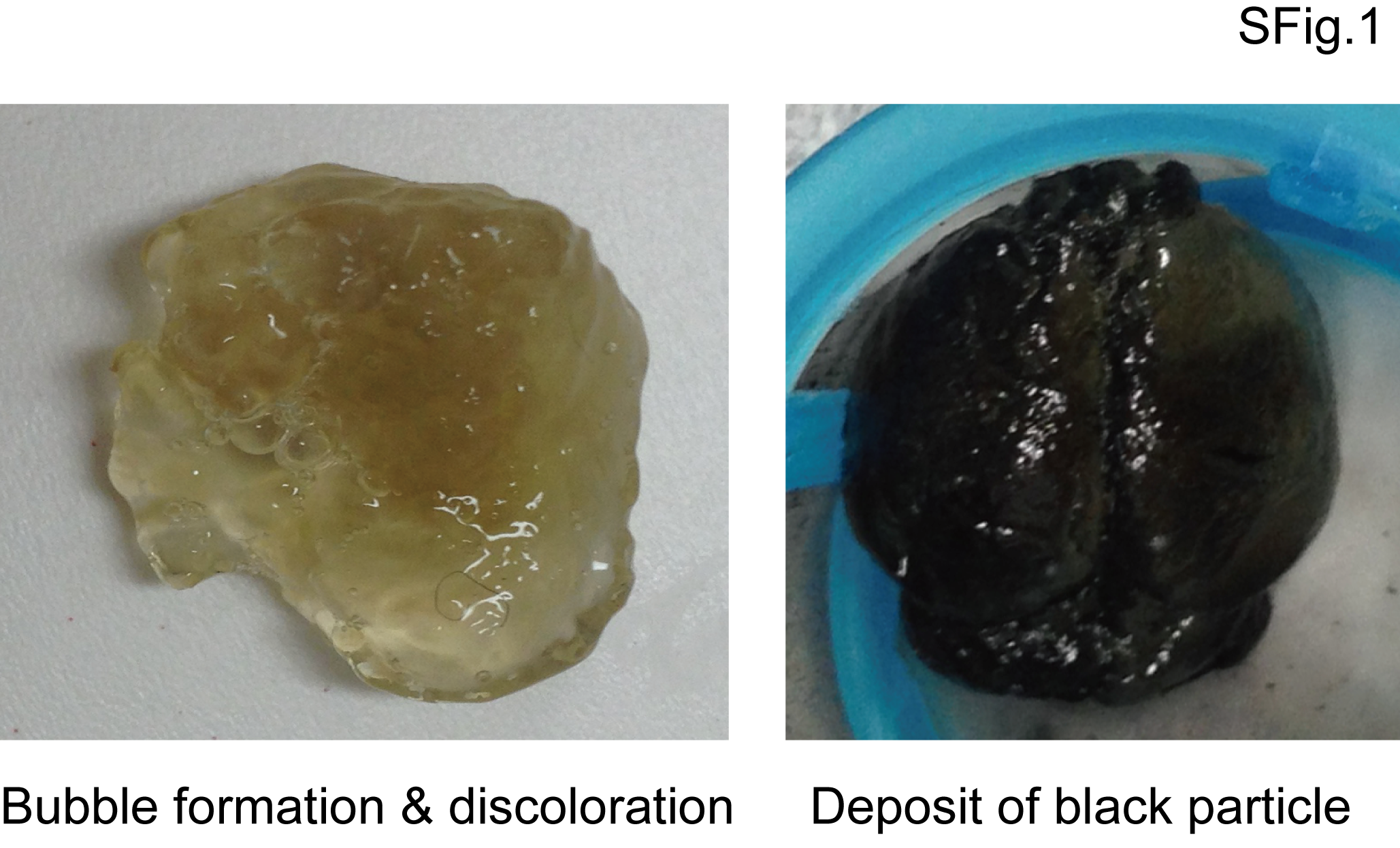

Supplement: Supplementary file 1 — Additional file 1: Figure that Problems of high voltage condition (30 voltage, 42°C) during CLARITY. (A) Bubble formation and discoloration in brain. (B) Deposit of black particle in brain. (TIFF 12 MB) [file 12861_2014_48_MOESM1_ESM.tiff]

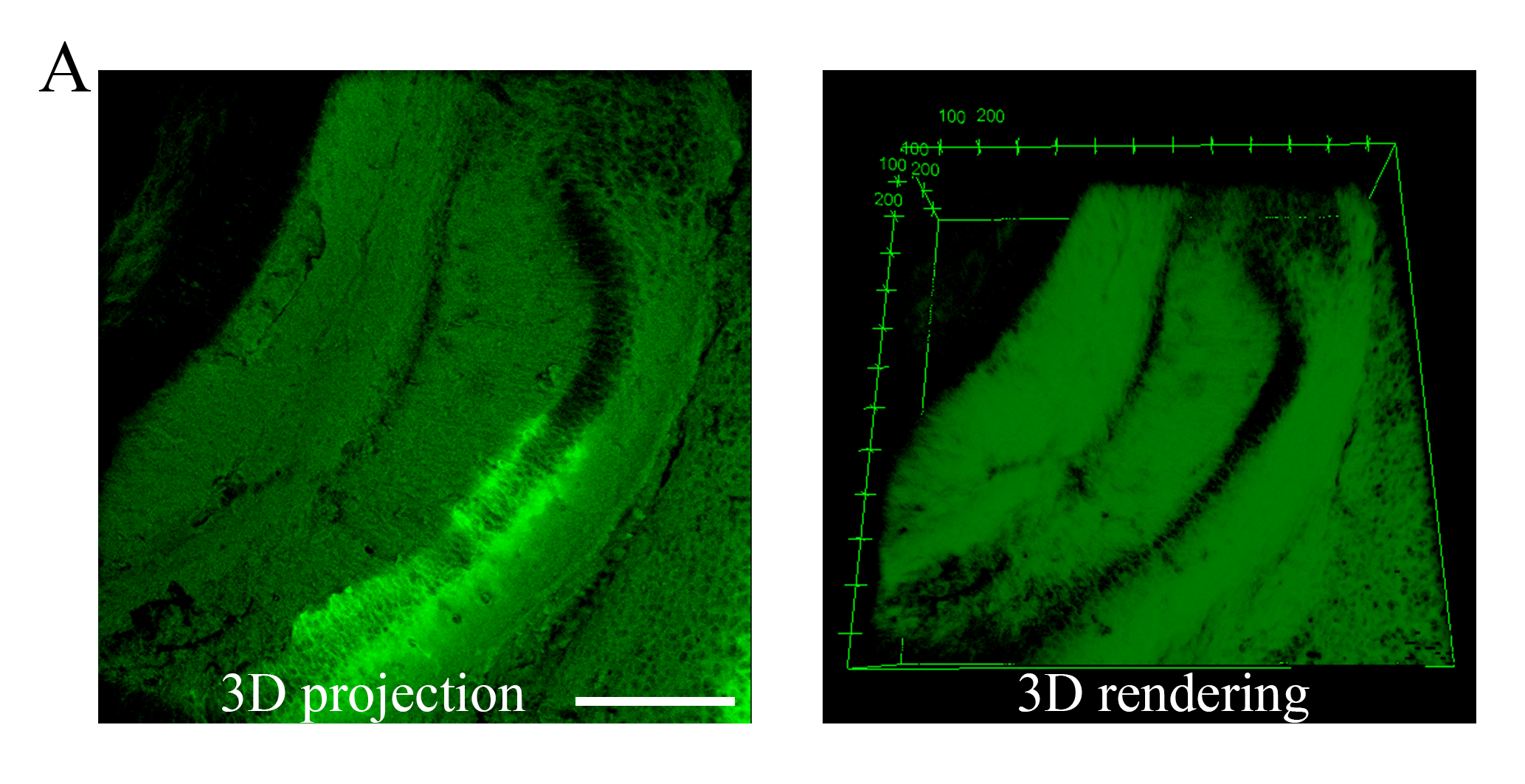

Supplement: Supplementary file 2 — Additional file 2: Figure that three-dimensional (3D) projection (left panel) and rendering (right panel) of clarified mouse brain immunostained for tau (green). Scale bar, 300 μm. (TIFF 6 MB) [file 12861_2014_48_MOESM2_ESM.tiff]
